# Breastfeeding difficulties and maternal–infant bonding at three months postpartum in a Turkish cohort

**DOI:** 10.3389/fped.2026.1863943

**Published:** 2026-08-18

**Authors:** Bulent Gunes, Ferit Dogan, Ahmet Guzelcicek, Aydin Bozkaya, S. Songul Yalcin

**Affiliations:** 1 Department of Pediatrics, Faculty of Medicine, Harran University, Sanliurfa, Turkey; 2 Department of Radiology, Faculty of Medicine, Harran University, Sanliurfa, Turkey; 3 Unit of Social Pediatrics, Department of Pediatrics, Faculty of Medicine, Hacettepe University, Ankara, Turkey

**Keywords:** breast feeding, infant care, maternal behavior, mother-child relations, postpartum period

## Abstract

**Background:**

Breastfeeding difficulties are common in the early postpartum period and have been hypothesized to negatively affect maternal–infant bonding. We aimed to investigate the relationship between breastfeeding problems and mother–infant bonding at 3 months postpartum in a sample of Turkish mothers.

**Methods:**

We conducted a cross-sectional observational study of 257 mothers of 3-month-old infants in Turkey. Participants completed a questionnaire including demographic and obstetric information, breastfeeding practices and difficulties, and the Postpartum Bonding Questionnaire (PBQ) to assess maternal–infant bonding. Bonding difficulty was defined as a PBQ total score >25. Bivariate analyses (chi-square or Fisher's exact tests) compared mothers with and without bonding difficulties, and a multivariable logistic regression model was used to identify independent predictors of bonding problems at 3 months postpartum.

**Results:**

Overall, 33 mothers (12.8%) met criteria for bonding difficulties at 3 months. A total of 144 mothers (56.0%) reported at least one breastfeeding problem during the first 3 months postpartum. In bivariate analyses, breastfeeding difficulties were more common among mothers with bonding difficulties than among those without (81.8% vs. 52.2%, *P* = .001). Mothers with bonding difficulties were also more likely to have higher education (81.8% vs. 54.9% with ≥ secondary education; *P* = .003), to be employed outside the home (21.2% vs. 8.9%; *P* = .06), and to have a female infant (66.7% vs. 38.8% female; *P* = .003). No significant differences were found by maternal age, parity, or feeding mode (exclusive breastfeeding vs. formula feeding, *P* = .64). In multivariable logistic regression, breastfeeding difficulties were independently associated with bonding impairment (adjusted odds ratio [OR] 3.5, 95% confidence interval [CI] 1.5–8.0, *P* = .003). Maternal higher education (adjusted OR 3.0, 95% CI 1.1–8.0, *P* = .031) and having a female infant (adjusted OR 3.1, 95% CI 1.2–7.5, *P* = .016) were also independent predictors; maternal employment showed a positive but non-significant association (adjusted OR 2.2, 95% CI 0.9–5.5, *P* = .081).

**Conclusions:**

In this Turkish sample, mothers who reported breastfeeding difficulties had higher odds of impaired maternal–infant bonding at 3 months postpartum. These findings support routine assessment of breastfeeding-related distress during postpartum care.

## Introduction

The establishment of a secure maternal–infant relationship in the early postpartum period is widely recognized as a determinant of long-term neurodevelopmental and psychosocial outcomes. Early bonding supports infant physiological regulation and emotional security and is also associated with maternal psychological well-being (1, 2). Disruptions in this process have been linked to postpartum depression, anxiety, and less favorable cognitive and socioemotional development in children (3). Identifying modifiable factors that may influence early bonding therefore remains a priority in perinatal research.

Breastfeeding is recommended by major health authorities, including the World Health Organization and the American Academy of Pediatrics, because of its established nutritional and immunological benefits (4). In addition to these benefits, breastfeeding has been proposed as a context that may support maternal–infant bonding. Skin-to-skin contact and the neuroendocrine effects of lactation, particularly oxytocin release, are thought to enhance maternal responsiveness and reduce stress (5). However, breastfeeding is not always straightforward. Many mothers experience early challenges such as nipple pain, latch difficulties, engorgement, or concerns about insufficient milk supply (6). These difficulties may be physically and emotionally burdensome and may affect maternal confidence and the quality of early mother–infant interactions (7).

The relationship between infant feeding and bonding remains complex. Earlier studies suggested that feeding method alone (breast versus formula) does not consistently determine bonding quality (8). More recent evidence indicates that the subjective experience of breastfeeding—especially when characterized by pain, frustration, or perceived inadequacy—may be more closely associated with maternal emotional outcomes than feeding mode itself (9). Higher breastfeeding self-efficacy has been associated with more positive maternal adaptation, whereas persistent difficulties have been linked to distress and vulnerability to bonding disturbances (9).

This distinction is particularly relevant in settings where breastfeeding is strongly normative. In Turkey, breastfeeding initiation rates are high, although exclusive breastfeeding often declines during the first months postpartum (10). In such a context, breastfeeding difficulties may carry additional emotional significance, particularly when they are perceived as not meeting cultural or familial expectations. Despite this, data from Turkish populations remain limited. Although some Turkish studies have examined breastfeeding attachment and validated maternal–infant bonding measures in this setting (10–12), few have specifically examined breastfeeding difficulties, rather than feeding type alone, in relation to maternal–infant bonding.

To address this gap, we evaluated the association between breastfeeding difficulties and maternal–infant bonding at three months postpartum in a cross-sectional sample of Turkish mothers. We hypothesized that mothers reporting breastfeeding difficulties would have higher odds of bonding impairment, independent of maternal demographic characteristics and infant factors.

## Methods

### Study design and setting

This cross-sectional observational study was conducted at the pediatric outpatient clinic of Harran University Faculty of Medicine Research and Application Hospital, a tertiary healthcare facility in southeastern Türkiye. Mothers were recruited during routine 3-month well-child visits or vaccination appointments between February 2026 and April 2026. The clinic serves both urban and surrounding rural populations.

### Participants and recruitment

Eligible participants were mothers aged 18 years or older with a healthy infant aged approximately 90 ± 15 days. Mothers were included if they were the primary caregiver and had breastfed or attempted to breastfeed postpartum, regardless of formula use. We excluded mothers of infants with major congenital anomalies or severe neonatal complications requiring prolonged neonatal intensive care unit (NICU) admission, as these circumstances involve mother–infant separation that may independently and substantially affect bonding. Infants born at a gestational age of less than 35 weeks were also excluded, given that prematurity at this degree is associated with NICU admission and disrupted early contact. Mothers of multiple-gestation pregnancies (twins or higher-order multiples) were excluded, as the caregiving dynamics and feeding experiences in these dyads differ substantially from singleton pregnancies. Mothers in single-parent households were not enrolled, as the absence of a partner may independently confound maternal bonding outcomes through differential levels of social support and caregiving stress. Mothers with known severe psychiatric disorders were similarly excluded.

During the study period, all mothers attending the clinic who met the eligibility criteria were consecutively invited to participate. Of 291 eligible mothers approached, 34 either declined participation or returned incomplete questionnaires and were not included, yielding a final analytic sample of 257 mothers (participation rate, 88.3%). Participation was voluntary, and mothers provided written informed consent before completing the questionnaire.

The required sample size was estimated *a priori* using a two-proportion test. Based on prior literature reporting a bonding difficulty prevalence of approximately 10%–15% and an anticipated absolute difference of 15–18 percentage points between mothers with and without breastfeeding difficulties (13), a minimum of approximately 220 participants was estimated to provide 80% statistical power at a two-sided alpha of 0.05. Accounting for a potential refusal or incomplete data rate of approximately 10%–15%, a target sample of 250–260 participants was established. The final enrolled sample of 257 mothers met this pre-specified target.

### Data collection

After consent, participants completed a structured questionnaire in a private setting. The questionnaire was administered in Turkish and underwent forward translation and pilot testing to ensure clarity. Research assistants were available to provide clarification if needed. The instrument included three domains: (1) socio-demographic and clinical characteristics, (2) breastfeeding practices and difficulties, and (3) maternal–infant bonding.

### Socio-demographic and clinical characteristics

Mothers reported age, education level, and employment status. Education was categorized as primary school or less (≤5 years) versus at least secondary education. Employment was classified as homemaker/unemployed versus employed (including those on maternity leave). Obstetric variables included parity (primiparous vs. multiparous) and mode of delivery (vaginal vs. cesarean section). Infant sex, gestational age, and birth weight were obtained from medical records. Mothers also reported whether the infant had experienced any significant health problems since birth, defined as conditions requiring medical evaluation, hospitalization, or specialist referral (e.g., jaundice requiring phototherapy, respiratory distress, feeding difficulties attributed to a medical diagnosis, or other acute illnesses). Infants who had required hospitalization after the newborn period were recorded separately, as postnatal hospitalization involving mother–infant separation was considered a potential confounding factor for bonding.

### Breastfeeding practices and difficulties

Feeding mode at 3 months was categorized as exclusive breastfeeding or any formula feeding (partial or exclusive). Mothers were asked whether breastfeeding was initiated within the first hour after birth. Pacifier use and bottle introduction (for expressed milk or formula) were recorded.

Breastfeeding difficulties were assessed using a predefined checklist of common early-lactation problems derived from established literature (6–8, 14): poor latch, nipple pain or cracking, engorgement, perceived insufficient or delayed milk supply, and infant refusal of the breast. For each item, mothers indicated whether the problem had occurred during the early postpartum period (first 4–6 weeks) and/or was ongoing at the 3-month assessment. These domains reflect the most frequently reported breastfeeding difficulties in observational studies and are consistent with the constructs assessed in validated lactation instruments. For the primary analysis, “any breastfeeding difficulty” was defined as the presence of at least one reported breastfeeding problem at any time during the first three months postpartum (early postpartum and/or ongoing at 3 months). Early difficulties (first 4–6 weeks) and current difficulties (at 3 months) were also recorded separately for descriptive analyses.

### Maternal–infant bonding assessment

Maternal–infant bonding was assessed using the Postpartum Bonding Questionnaire (PBQ), developed by Brockington and colleagues (15). The PBQ consists of 25 items measuring maternal emotional responses toward the infant, rated on a Likert scale, with higher scores indicating greater bonding disturbance. The Turkish version (Doğum Sonrası Bağlanma Ölçeği, DSBÖ) has been validated in Turkish mothers: prior work reported acceptable internal consistency and a coherent factor structure, together with expected correlations between PBQ scores and postpartum depressive-symptom measures, supporting the construct validity of the instrument in this population (11, 16). In the present sample, internal consistency of the PBQ total score was acceptable (Cronbach's *α* = 0.79).

Bonding difficulty was defined using the recommended cutoff of a PBQ total score >25 (13). Mothers exceeding this threshold were classified as having impaired bonding. This total-score threshold was derived from Brockington's original (predominantly Western) validation work; a Turkish-population-specific cutoff has not been formally established, and this is acknowledged as a limitation.

### Statistical analysis

Data were analyzed using SPSS version 26.0 (IBM Corp., Armonk, NY). Continuous variables are presented as means ± standard deviations and categorical variables as frequencies and percentages. Bivariate comparisons between mothers with and without bonding difficulties were performed using chi-square tests (or Fisher's exact tests when appropriate) for categorical variables and independent-samples t-tests for continuous variables.

Variables associated with bonding status at *P* < 0.25 in bivariate analyses were considered for entry into the multivariable logistic regression model. This liberal screening threshold follows the purposeful-selection recommendation of Hosmer et al. and of Bursac et al., who caution that the conventional *P* < 0.05 criterion can fail to identify variables that become important confounders once other covariates are present; the more permissive threshold reduces the risk of omitting relevant confounders in a moderately sized sample (17, 18). For every categorical predictor, the reference category was as follows: no breastfeeding difficulty (for the breastfeeding-difficulty exposure), primary education or less (for maternal education), homemaker/unemployed (for maternal employment), and male infant (for infant sex). Because the outcome comprised only 33 events, the number of covariates was constrained to preserve an adequate events-per-variable ratio and to limit overfitting; the final model was therefore restricted to the primary exposure (any breastfeeding difficulty) and three pre-specified covariates of primary clinical interest (maternal education, employment status, and infant sex). Mode of delivery, although strongly associated with bonding status in bivariate analysis and meeting the screening threshold, was not entered into the final model for this reason and is highlighted as a target for future adequately powered studies. Infant health problems could not be entered because no infant health problem occurred among mothers in the bonding-difficulty group, producing complete separation that precludes stable maximum-likelihood estimation; this variable was therefore examined descriptively only. Adjusted odds ratios (ORs) and 95% confidence intervals (CIs) were calculated. Model fit was assessed using the Hosmer–Lemeshow test, and multicollinearity among the retained covariates was evaluated using variance inflation factors (VIF), all of which were below 5. All tests were two-tailed, with *P* < 0.05 considered statistically significant. Missing data were minimal (≤1.2% for any single variable) and handled by pairwise exclusion. Pacifier and bottle use, although significantly associated with bonding status in bivariate analyses, were not entered as independent covariates in the multivariable model, as both were considered closely collinear with the primary breastfeeding-difficulty exposure (their introduction commonly follows or accompanies feeding-related challenges at the breast); including them alongside the primary exposure would have compromised model parsimony and interpretability without adding independent explanatory value.

## Results

### Participant characteristics

A total of 257 mothers and their 3-month-old infants were included. The mothers had a mean age of 28.9 ± 5.4 years (range 18–42 years); 37.7% were primiparous and 62.3% multiparous. At least secondary education was reported by 58.4% of participants, whereas 41.6% had primary education or less. Most mothers (89.5%, *n* = 230) were homemakers, and 10.5% (*n* = 27) were in paid employment (most on maternity leave at 3 months). Delivery was by cesarean section in 43.6% (*n* = 112) and vaginal in 56.4% (*n* = 145). About 8% of infants were late preterm (34–36 weeks); no infant was born before 34 weeks. Infant boys comprised 57.6% (*n* = 148) and girls 42.4% (*n* = 109) of the sample (Table 1).

**Table 1:** Demographic and clinical characteristics of mothers with and without maternal–infant bonding difficulties at 3 months postpartum.

Characteristic	Category	No bonding difficulty (*n* = 224)	Bonding difficulty (*n* = 33)	*P* Value
Maternal age	Mean ± SD, years	28.8 ± 5.5	29.6 ± 5.1	.400*
Age group	< 25 years	56 (25.0%)	9 (27.3%)	.268
	25–34 years	121 (54.0%)	21 (63.6%)	
	≥ 35 years	47 (21.0%)	3 (9.1%)	
Parity	Primiparous (first child)	85 (37.9%)	12 (36.4%)	.861
	Multiparous (≥2 children)	139 (62.1%)	21 (63.6%)	
Maternal education	Primary or less (≤5 years)	101 (45.1%)	6 (18.2%)	.**003**
	≥ Secondary (middle school+)	123 (54.9%)	27 (81.8%)	
Maternal employment	Homemaker/unemployed	204 (91.1%)	26 (78.8%)	.060
	Employed (working mother)	20 (8.9%)	7 (21.2%)	
Father's education	Primary or less	34 (15.2%)	3 (9.1%)	.437
	≥ Secondary	190 (84.8%)	30 (90.9%)	
Mode of delivery^a^	Vaginal delivery	137 (61.2%)	8 (24.2%)	**<**.**001**
	Cesarean section	87 (38.8%)	25 (75.8%)	
Infant sex	Male	137 (61.2%)	11 (33.3%)	.**003**
	Female	87 (38.8%)	22 (66.7%)	
Infant birth order	First-born	82 (36.6%)	15 (45.5%)	.328
(overlaps with parity)	Second-born or later	142 (63.4%)	18 (54.5%)	
Infant gestational age^b^	Late preterm (34–36 wks)	17 (7.6%)	3 (9.1%)	.809
(at birth)	Early term (37–38 wks)	97 (43.3%)	16 (48.5%)	
	Full term (39–41 wks)	107 (47.8%)	14 (42.4%)	
Infant size at birth	Small for GA (SGA)	17 (7.6%)	0 (0%)	.194
(Birth weight vs. GA)	Appropriate (AGA)	157 (70.1%)	27 (81.8%)	
	Large for GA (LGA)	50 (22.3%)	6 (18.2%)	
Infant health problems	None since birth	194 (86.6%)	33 (100%)	.019**
(0–3 months)	Yes (any significant issue)	30 (13.4%)	0 (0.00%)	

GA, gestational age; SGA/AGA/LGA, small/appropriate/large for gestational age (relative to population birthweight percentiles).

a For mode of delivery, Fisher's exact test was used because of small expected cell counts in the bonding-difficulty group.

b Gestational age was unavailable for 3 mothers (1.3%) in the no-bonding-difficulty group.

*P value for maternal age is from independent *t*-test comparing mean ages. All other *P* values from chi-square or Fisher's exact tests, as appropriate.

**P value from Fisher's exact test due to low expected cell counts (including a zero cell).

Bold values indicate statistically significant results (*p* < 0.05) for maternal education, mode of delivery, infant sex, and infant health problems.

### Prevalence of bonding difficulties

Based on the PBQ (total score >25), 33 of 257 mothers (12.8%) were classified as having bonding difficulties at 3 months postpartum; the remaining 224 (87.2%) scored in the normal range. Elevated scores generally reflected reduced enjoyment of or emotional connection with the infant, sometimes accompanied by anger, anxiety, or disappointment during interaction. No mother endorsed active thoughts of harming her baby (the most extreme PBQ items).

### Breastfeeding practices and difficulties

At 3 months, 162 mothers (63.0%) were exclusively breastfeeding, while 95 (37.0%) had introduced formula as a supplement or as the primary mode; of these 95, only 18 (7.0% of the total sample) had switched entirely to formula. Nearly all mothers (96%) had attempted to breastfeed from birth, and 139 (54.1%) initiated breastfeeding within the first hour, whereas 118 (45.9%) did not (Table 2).

**Table 2:** Breastfeeding and infant feeding-related factors among mothers with and without bonding difficulties at 3 months. The table shows the distribution of feeding practices and breastfeeding problems in each group, with corresponding chi-square or Fisher's exact *P* values.

Feeding factor	Category	No bonding difficulty (*n* = 224)	Bonding difficulty (*n* = 33)	*P* value
Feeding mode at 3 months	Exclusive breastfeeding	140 (62.5%)	22 (66.7%)	.643
	Partial/Formula feeding	84 (37.5%)	11 (33.3%)	
Any breastfeeding difficulty (early and/or current)*	No	107 (47.8%)	6 (18.2%)	**.** **001**
	Yes	117 (52.2%)	27 (81.8%)	
Early postpartum difficulty (first 4–6 weeks)	No	107 (47.8%)	6 (18.2%)	.**001**
	Yes	117 (52.2%)	27 (81.8%)	
Current difficulty at 3 months	No	114 (50.9%)	10 (30.3%)	.**027**
	Yes	110 (49.1%)	23 (69.7%)	
Specific BF issue: Poor latch	No	203 (90.6%)	30 (90.9%)	1.000
	Yes	21 (9.4%)	3 (9.1%)	
Specific BF issue: Nipple pain	No	182 (81.3%)	24 (72.7%)	.252
	Yes	42 (18.8%)	9 (27.3%)	
Specific BF issue: Engorgement	No	215 (96.0%)	30 (90.9%)	.189
	Yes	9 (4.0%)	3 (9.1%)	
Specific BF issue: Low milk	No	170 (75.9%)	21 (63.6%)	.132
	Yes	54 (24.1%)	12 (36.4%)	
Specific BF issue: Baby refused breast	No	214 (95.5%)	33 (100%)	.370
	Yes	10 (4.5%)	0 (0%)	
Pacifier use by 3 months	No (never used)	91 (40.6%)	22 (66.7%)	.**005**
	Yes	133 (59.4%)	11 (33.3%)	
Bottle feeding by 3 months	No (no bottle introduced)	102 (45.5%)	9 (27.3%)	.**048**
	Yes	122 (54.5%)	24 (72.7%)	
Fed in first hour after birth	No	103 (46.0%)	15 (45.5%)	.955
	Yes	121 (54.0%)	18 (54.5%)	

BF, breastfeeding; SD, standard deviation; PBQ, postpartum bonding questionnaire.

* Early postpartum difficulties refer to breastfeeding problems occurring within the first few weeks after birth, as recalled by the mother at the 3-month assessment. For analytical purposes, “any breastfeeding difficulty” was defined as the presence of at least one reported breastfeeding problem during the first three months postpartum (i.e., early postpartum and/or ongoing at 3 months). In this sample, all mothers who reported ongoing difficulties at 3 months had also experienced difficulties in the early postpartum period; therefore, the numerical distribution of “any difficulty” corresponds to that of early postpartum difficulty. Percentages are calculated within column totals (No bonding difficulty: *n* = 224; Bonding difficulty: *n* = 33).

*P* values were derived from chi-square or Fisher's exact tests, as appropriate; values in bold indicate statistical significance at *P* < .05, and values in bold indicate trend-level significance (0.05 ≤ *P* < .10).

Overall, 144 mothers (56.0%) reported at least one breastfeeding problem during the first 3 months; 133 (51.8%) reported a current problem at 3 months and 144 (56.0%) reported early difficulties in the initial postpartum weeks. The most frequently reported specific problems were perceived delayed or inadequate milk supply (25.7%), nipple pain or cracking (19.8%), poor latch (9.3%), engorgement (4.7%), and infant refusal of the breast (3.9%) (Table 2). Pacifiers had been used by 144 infants (56.0%), and a bottle (for expressed milk or formula) had been introduced by 146 mothers (56.8%) (Table 2).

### Bivariate associations with bonding difficulties

The primary exposure showed the expected association: among the 33 mothers with bonding difficulties, 27 (81.8%) had experienced at least one breastfeeding difficulty (early or ongoing), compared with 117 of 224 (52.2%) mothers with normal bonding (*P* = .001). Considering only current status at 3 months, 23 of 33 (69.7%) bonding-impaired mothers reported an ongoing breastfeeding problem, versus 110 of 224 (49.1%) mothers with normal bonding (*P* = .027). Among specific problems, mothers with bonding difficulties more often reported perceived low or delayed milk supply (36.4% vs. 24.1%; *P* = .132) and nipple pain (27.3% vs. 18.8%; *P* = .252), although these individual comparisons did not reach significance (Table 2).

Feeding mode alone was not associated with bonding status. Exclusive breastfeeding was similarly frequent in the bonding-difficulty and normal-bonding groups (66.7% vs. 62.5%; *P* = .64); among the 33 bonding-impaired mothers, 22 (66.7%) were exclusively breastfeeding and 11 (33.3%) were partially or fully formula feeding, a distribution comparable to that of mothers without bonding difficulties (Table 2).

Several demographic and infant factors were also associated with bonding status. Higher maternal education was more common among mothers with bonding difficulties (81.8% vs. 54.9% with ≥ secondary education; *P* = .003); only 6 of 107 mothers with primary education or less (5.6%) had bonding difficulties. Bonding difficulties were also more frequent among employed mothers than homemakers: 21.2% of bonding-impaired mothers versus 8.9% of those without bonding difficulties were employed (*P* = .06), equivalent to a bonding-difficulty rate of 25.9% (7/27) among employed mothers versus 11.3% (26/230) among homemakers. Infant sex was strongly associated with bonding: 22 of 33 bonding-impaired mothers (66.7%) had a female infant, versus 38.8% in the normal-bonding group (*P* = .003); expressed as a rate, 20.2% of mothers of girls versus 7.4% of mothers of boys had bonding difficulties (Table 1).

Maternal age (*P* = .40), age category (*P* = .37), and parity (*P* = .86) were not associated with bonding status. Cesarean delivery was significantly more frequent among mothers with bonding difficulties (75.8% vs. 38.8%; *P* < .001, Fisher's exact test); because of the limited number of events, it was not entered into the multivariable model (see Methods) and warrants dedicated investigation in future studies. Pacifier use was less common among bonding-impaired mothers (33.3% vs. 59.4%; *P* = .005), whereas bottle introduction was more common (72.7% vs. 54.5%; *P* = .048). Notably, none of the 30 mothers who reported a significant infant health problem were in the bonding-difficulty group (0/30 vs. 33/227; *P* = .019, Fisher's exact test); this complete separation both precluded inclusion of the variable in the regression and likely reflects the small subgroup size (Tables 1, 2).

**Figure 1:**
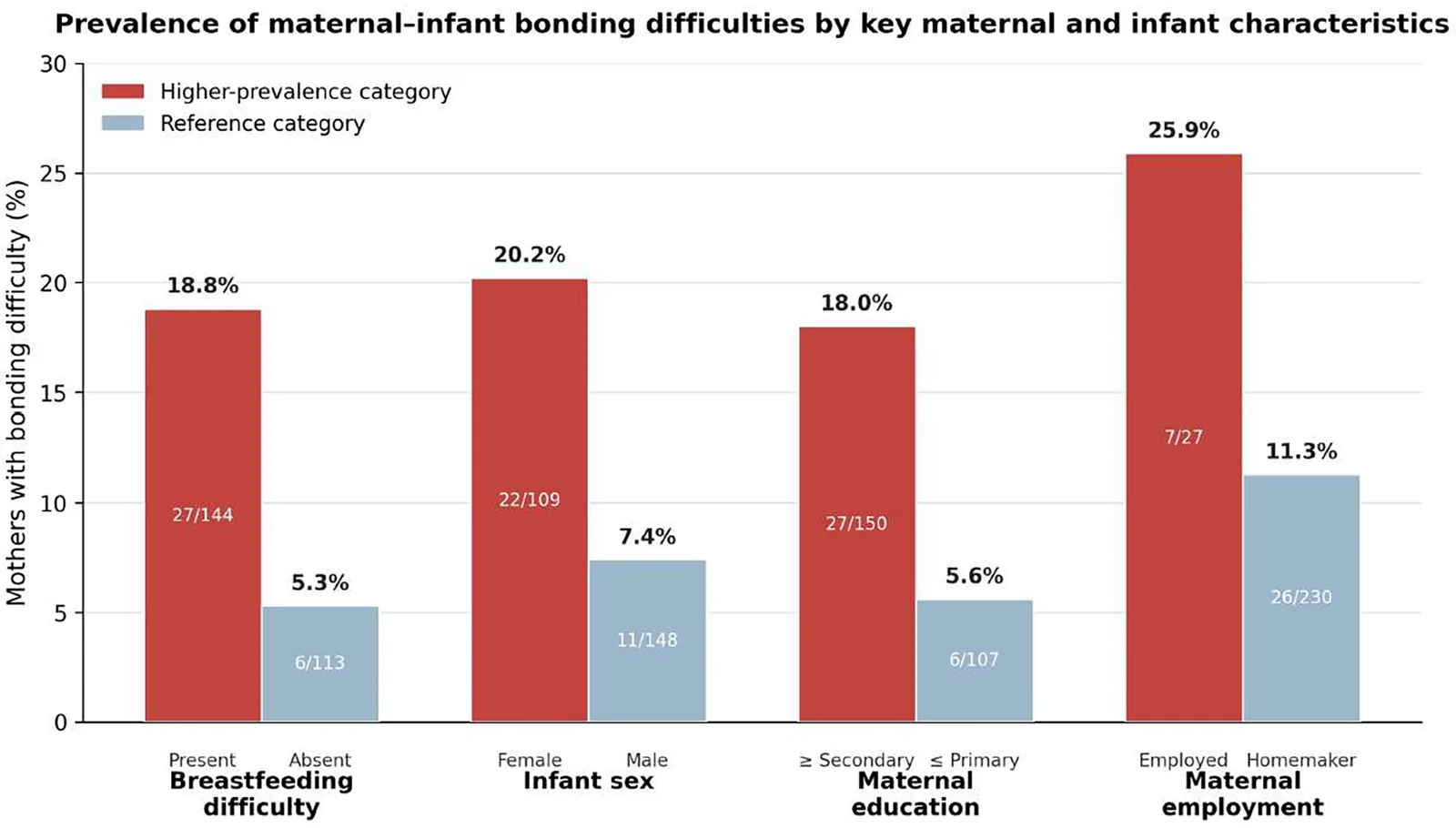
Prevalence of maternal–infant bonding difficulties according to key maternal and infant characteristics at 3 months postpartum. For each factor, paired bars show the percentage of mothers with bonding difficulties in the higher-prevalence category and the reference category, with the underlying counts (n with bonding difficulty/N in category). Rates were higher among mothers with breastfeeding difficulties (18.8% [27/144] vs. 5.3% [6/113]), mothers of female infants (20.2% [22/109] vs. 7.4% [11/148]), mothers with at least secondary education (18.0% [27/150] vs. 5.6% [6/107]), and employed mothers (25.9% [7/27] vs. 11.3% [26/230]).

The prevalence of bonding difficulties across the principal maternal and infant characteristics is summarized in Figure 1.

### Multivariable logistic regression

The final multivariable logistic regression model included four covariates: any breastfeeding difficulty (reference: none), maternal education (reference: ≤ primary), maternal employment (reference: homemaker), and infant sex (reference: male). The model was statistically significant overall (model *χ*^2^ = 29.1, *P* < .001) with adequate fit (Hosmer–Lemeshow *P* = .45) (Table 3).

**Table 3:** Multivariable logistic regression analysis predicting maternal–infant bonding difficulties at 3 months postpartum (*n* = 257).

Predictor variable	Adjusted OR (95% CI)	*P* Value
Breastfeeding difficulties (Yes vs. No)	3.5 (1.5–8.0)	.**003**
Maternal higher education (≥ secondary vs. ≤primary)	3.0 (1.1–8.0)	.**031**
Maternal employment (Yes vs. No)	2.2 (0.9–5.5)	.081
Infant female sex (Female vs. Male)	3.1 (1.2–7.5)	.**016**

Reference categories: no breastfeeding difficulty; primary education or less; homemaker/unemployed; male infant. Model *χ*^2^= 29.1, *P* < .001; Hosmer–Lemeshow *P* = .45.

OR, odds ratio; CI, confidence interval.

Bold *P* values denote *P* < .05.

After adjustment, breastfeeding difficulties remained associated with bonding impairment in the prespecified adjusted model (adjusted OR 3.5, 95% CI 1.5–8.0, *P* = .003), corresponding to more than three-fold higher odds of impaired bonding. Higher maternal education (adjusted OR 3.0, 95% CI 1.1–8.0, *P* = .031) and female infant sex (adjusted OR 3.1, 95% CI 1.2–7.5, *P* = .016) were also independent predictors. Maternal employment showed a positive point estimate (adjusted OR 2.2, 95% CI 0.9–5.5, *P* = .081), but because the 95% CI crossed 1 this association did not reach statistical significance; accordingly, no firm conclusion about an independent effect of employment can be drawn, and the estimate should be interpreted with caution. The variable was retained given its clinical relevance and its bivariate association.

Parity did not meet the screening threshold and was not considered further. Mode of delivery, although significantly associated with bonding status in bivariate analysis, was not entered into the final model in order to preserve an adequate events-per-variable ratio (see Methods); its independent contribution should be evaluated in larger studies. Overall, breastfeeding difficulties remained associated with bonding impairment in the prespecified adjusted model.

## Discussion

In this cross-sectional study of Turkish mothers of 3-month-old infants, mothers who reported breastfeeding difficulties were significantly more likely to report impaired maternal–infant bonding, and this association persisted after adjustment for key maternal and infant characteristics. Quantitative data from Turkish cohorts on breastfeeding difficulties and early bonding remain limited. The present findings are consistent with evidence suggesting that bonding may be more closely related to the quality of the feeding experience—particularly whether it is experienced as manageable or persistently stressful—than to feeding mode alone (13, 19).

Breastfeeding difficulties were associated with approximately three-fold higher odds of impaired bonding in the adjusted model (adjusted OR 3.5). This is consistent with evidence from other settings that negative breastfeeding experiences may influence maternal emotional well-being and feelings toward the infant (20, 21). When breastfeeding is painful, exhausting, or perceived as unsuccessful, mothers may experience heightened stress, lowered confidence, and feelings of inadequacy, which can make emotional closeness more difficult (22); conversely, when breastfeeding proceeds smoothly, a sense of mastery may support bonding (23).

Our study extends these observations to a setting where breastfeeding is highly normative. Nearly all mothers initiated breastfeeding and more than half were exclusively breastfeeding at 3 months, so our comparison largely reflects successful breastfeeding versus breastfeeding continued in the context of difficulties, rather than breastfeeding versus not breastfeeding. In this context, feeding mode alone (exclusive or partial breastfeeding versus formula feeding) did not differentiate bonding outcomes, supporting the view that the mode of feeding is not itself the principal determinant (23). This aligns with prior work indicating that mothers’ subjective feeding experience—confidence, stress, satisfaction—may be more closely related to bonding than feeding method (20, 24).

Causality cannot be inferred from a cross-sectional design, and reverse causation is plausible: mothers already struggling with bonding or with emerging mood symptoms may perceive breastfeeding as more difficult or may have fewer resources to manage routine challenges (24–26). Although we adjusted for several confounders, residual confounding by unmeasured psychosocial variables remains possible, and longitudinal studies are needed to clarify directionality. Nonetheless, the persistence of the association after adjustment suggests that breastfeeding difficulties may have an effect on bonding that is at least partly independent of measured covariates, consistent with prior studies that identified breastfeeding difficulties as a risk factor for less favorable bonding even after accounting for depressive symptoms (13).

A notable finding was that exclusive breastfeeding versus formula feeding was not associated with differences in bonding, consistent with empirical work reporting no consistent association between feeding mode alone and mother–infant bonding once contextual factors are considered (27, 28). These findings do not support the assumption that formula feeding is inherently associated with poorer bonding. Rather, the quality of interaction during feeding—physical closeness, eye contact, sensitivity to infant cues, and responsive caregiving—may be more relevant to bonding than milk source (29).

We also observed that mothers who never used a pacifier had higher rates of bonding problems, whereas bonding difficulties were more common among mothers who had introduced a bottle. Pacifier use has been associated with reduced maternal stress and more positive interactions, possibly by soothing infants and reducing maternal exhaustion (30, 31); mothers who avoided pacifiers may have done so because of breastfeeding advice or preference, or their infants may have been harder to console (32, 33). Bottle introduction may reflect practical needs (e.g., impending return to work) or clinical advice to supplement (34), and reverse causality is again possible, as mothers who feel less connected may be more willing to delegate feeding (34, 35). These associations warrant further qualitative and longitudinal study.

An unexpected result was that higher maternal education was associated with increased bonding difficulties, with roughly three-fold higher odds compared with less-educated mothers (36, 37). Although counterintuitive, several explanations are plausible. In our sample, education was closely linked to employment—many higher-educated mothers were working, whereas virtually none of the low-education mothers were employed—so education may partly proxy the pressures of balancing work with early motherhood. In Turkey, statutory maternity leave spans approximately 16 weeks (38), coinciding with the 3-month postpartum period for many mothers; anticipation of, or recent return to, work may contribute to strain, guilt, and reduced time with the infant (39, 40). Higher education may also be associated with elevated expectations regarding breastfeeding, parenting competence, and the “ideal” bonding experience (41); when these are unmet, mothers may appraise their experience more negatively or more readily endorse PBQ items reflecting distress (42). Differential reporting is also possible, and stronger extended-family support among less-educated homemaker mothers may reduce stress and facilitate bonding (43); because social support was not directly measured, this remains speculative.

Maternal employment showed a consistent trend toward higher bonding difficulties. Although this did not remain statistically significant in the adjusted model (likely reflecting limited power, with a wide confidence interval crossing 1), the point estimate—more than twice the odds—may carry clinical relevance and is compatible with the possibility that early separation, role strain, and the cognitive load of managing work and infant care may, in some mothers, be accompanied by reduced emotional attunement (39, 43, 44). This should not be read as a universal effect of employment; many employed mothers bond well. Rather, it points to a subgroup that may benefit from counseling around role transitions, workplace flexibility, and strategies to protect mother–infant closeness (45–47). Overall, education and employment appear to contribute jointly, potentially through heightened expectations, time constraints, or tension between professional and maternal identities (48, 49), and may be amplified by cultural expectations that mothers prioritize infant caregiving in the early months.

We also found a strong association between having a female infant and bonding difficulties. In some societies, subtle son preference can influence postpartum experiences and family support following the birth of a girl, which could affect maternal emotional state and perceptions of the mother–infant relationship. Some studies report higher postpartum depression in mothers of female infants in settings where male children are culturally favored (50, 51); if such a mechanism were present here (depression was not measured), it could contribute to elevated PBQ scores. Infant sex should not be construed as causal; any association likely operates through psychosocial pathways shaped by family expectations, cultural context, and maternal adjustment, and the finding could also reflect residual confounding or sampling variation (52, 53).

Infant health problems showed a counterintuitive distribution: none of the mothers of infants with reported health issues were in the bonding-problem group. Heightened caregiving demands and perceived vulnerability may intensify protective feelings and mobilize support, buffering bonding difficulties; however, given the small subgroup (*n* = 30) and the absence of any bonding problems within it, this finding should be interpreted cautiously and may reflect limited power.

Our findings are consistent with prior research showing that feeding method alone may not determine bonding, whereas breastfeeding success, self-efficacy, or satisfaction are more closely linked to bonding outcomes (12, 13, 54, 55). Notably, Brockington et al. reported that breastfeeding problems were more common among mothers with bonding disorders, independent of depression (15); even without direct measurement of depression, breastfeeding difficulties remained a prominent correlate of bonding problems in our data. The Turkish validity and reliability of the PBQ/DSBÖ have been demonstrated, but the direct linkage between breastfeeding difficulties and PBQ scores has received little emphasis in Turkish cohorts, which our study specifically addresses (11, 16).

These results have practical implications for postpartum care. Clinicians and allied health professionals (obstetricians, pediatricians, midwives, and lactation consultants) should be attentive to mothers reporting significant breastfeeding pain, persistent latch problems, or high feeding-related anxiety, as this group may be at heightened risk for bonding difficulties; brief screening or structured conversations about the mother's feelings toward the baby may help identify concerns early, and timely lactation support may reduce feeding-related distress and help maintain positive mother–infant interaction. When breastfeeding remains difficult despite adequate support, nonjudgmental guidance around feeding alternatives is appropriate, the consistent message being a positive, supported feeding relationship—at breast or by bottle—in which mothers feel competent and emotionally available. For higher-educated and working mothers, counseling that addresses expectations and role transitions may be particularly helpful, and the association with infant sex highlights the need for sensitive, culturally informed psychosocial support where gender expectations may influence the postpartum experience.

### Strengths and limitations

This study examines an understudied aspect of early motherhood—breastfeeding *difficulties* rather than feeding mode alone—in a Turkish context, contributing cultural diversity to a largely Western literature. The sample size (*n* = 257) was adequate for multivariable analysis, participants were recruited in a real-world clinical setting, bonding was assessed with a validated instrument (PBQ/DSBÖ), and breastfeeding difficulties were captured across multiple specific domains.

Several limitations apply. The cross-sectional design precludes causal inference; breastfeeding difficulties and bonding were assessed at the same time point, so reverse causality or bidirectional effects cannot be excluded. Maternal mental health, particularly postpartum depression and anxiety, was not directly measured, leaving open the possibility of residual confounding. Both breastfeeding problems and bonding were self-reported, introducing potential recall and social-desirability bias. We did not record whether mothers had received formal lactation support, which may modify both feeding difficulties and emotional outcomes and represents a potential unmeasured confounder. The PBQ total-score cutoff of >25 was derived in predominantly Western validation samples; a Turkish-population-specific threshold has not been established, and misclassification of bonding status cannot be excluded. Because a purposeful-selection procedure was used, some degree of model-selection bias and overfitting is possible, so effect estimates should be interpreted with appropriate caution. Because mode of delivery was significantly associated with bonding status but was not included in the final multivariable model owing to the limited number of outcome events, residual confounding by delivery mode cannot be excluded. Multiple bivariate comparisons were performed without formal adjustment for multiple testing, so some univariate associations may reflect type I error. Generalizability may be limited, as the single-center sample may not reflect all regional and sociocultural variations within Turkey. As this study was designed as a cross-sectional assessment of the mother-infant bond at three months postpartum, breastfeeding data at six months were not available for the cohort in question; this constitutes a limitation, and studies with a six-month follow-up period are needed. Moreover, given that legal maternity leave in Turkey is limited to approximately 16 weeks (38), this policy context suggests that maternal bonding—particularly among highly educated and working mothers—may be diminished due to maternal stress and the challenges associated with returning to work. Finally, the single time-point assessment did not allow evaluation of longitudinal trajectories; because breastfeeding rates remain high in the first trimester and often decline thereafter, future studies extending follow-up to 6 months and beyond would help clarify whether resolution of feeding problems improves bonding over time.

Despite these limitations, the findings highlight a consistent association between breastfeeding-related distress and early bonding difficulties, emphasizing the clinical importance of addressing mothers’ feeding experiences within postpartum care.

## Conclusion

In this cross-sectional study of Turkish mothers at 3 months postpartum, breastfeeding difficulties (not feeding mode alone) were associated with maternal–infant bonding problems in the prespecified adjusted model. These findings suggest that the quality and emotional burden of the feeding experience may be more relevant for early bonding than whether a mother breastfeeds or formula feeds. Although causality cannot be established, the results support identifying and addressing breastfeeding-related distress as part of comprehensive postpartum care. Integrating lactation support with attention to the mother–infant relationship may help promote both feeding success and healthier early bonding outcomes.

## Data Availability

The original contributions presented in the study are included in the article/Supplementary Material, further inquiries can be directed to the corresponding author.
